# Complex Regional Pain Syndrome: Thalamic GMV Atrophy and Associations of Lower GMV With Clinical and Sensorimotor Performance Data

**DOI:** 10.3389/fneur.2021.722334

**Published:** 2021-09-22

**Authors:** Martin Domin, Sebastian Strauss, James H. McAuley, Martin Lotze

**Affiliations:** ^1^fMRI Unit, Diagnostic Radiology and Neuroradiology, University Medicine Greifswald, Greifswald, Germany; ^2^Neurology, University Medicine Greifswald, Greifswald, Germany; ^3^NeuRA and the School of Health Sciences, University of New South Wales, Sydney, NSW, Australia

**Keywords:** CRPS, chronic pain, neuropathic pain, gray matter volume, thalamus, ACC, insula

## Abstract

Results on gray matter alterations in complex regional pain syndrome (CRPS) showed heterogeneous findings. Since CRPS is a rare disease, most studies included only small and heterogeneous samples resulting in a low reliability of findings between studies. We investigated 24 CRPS patients with right upper limb affection in the chronic stage of disease using structural MRI and clinical testing. We focused on gray matter volume (GMV) alterations of the brain in comparison to 33 age matched healthy controls, their association to clinical characteristics (duration of pain syndrome and pain intensity ratings) and sensorimotor performance (finger dexterity and spatiotactile resolution). When applying an explorative whole brain analysis CRPS patients showed lower GMV in the bilateral medial thalamus. No other areas showed a relevant GMV difference for the group comparisons. When applying a region of interest driven approach using anatomical masks of the thalamus, ACC/mPFC, putamen, and insula we found relevant associations of clinical and behavioral data in ACC and insula. Whereas, the GMV in ACC showed negative associations with pain intensity and CRPS duration, the GMV of the left posterior insula was negatively associated with sensorimotor performance of the affected hand side. Overall, our results are in accordance to results of others describing a thalamic reduction of GMV in patients with neuropathic pain and are also in accordance with associations of pain intensity and duration with reduced ACC in general in patients with chronic pain syndromes. Sensorimotor performance seems to be related to posterior insula GMV reduction, which has not been described yet for other patient groups.

## Introduction

### GMV-Alterations in Chronic Pain

Chronic pain has a substantial impact on quality of life of patients and their families. With a prevalence of 20% (1) it also represents a major socio-economic challenge. By definition, chronic pain lasts for more than 12 weeks and importantly does not depend on sustained physical damage, i.e., may be maintained by alterations in the central nervous system (2). For structural alterations in patients suffering from various chronic pain conditions several meta-analyses [e.g., (3, 4)] have consistently described decreased gray matter volume in the medial prefrontal cortex (mPFC) and the anterior cingulate cortex (ACC) of the brain. For chronic low back pain (CLBP), as the most frequently occuring chronic pain syndrome, loss of gray matter volume (GMV) has been described for the medial prefrontal cortex, the anterior (ACC) and midcingulate (MCC) cortex and anterior insula [e.g., (5, 6)], but also for the thalamus [e.g., (7)].

Besides CLBP, neuropathic facial pain such as trigeminal neuralgia showed robust effects of GMV decrease in bilateral ACC (8, 10), insula (8) but also a reduction in thalamic GMV especially for those areas which are involved in sensorimotor processing (medial parts of the thalamus) (8, 9). Furthermore, since less pronounced pain intensity such as temporomandibular disorder (TMD) showed only marginal effects in ACC/mPFC GMV an association of GMV loss and pain severity has been assumed (11).

When using large samples, especially general population-based cohorts, the inter-individual random noise in the variance is significantly reduced, resulting in reliable and robust statistical testing, including correction, for e.g., multiple comparisons (12).

### CRPS Epidemiology and Characteristics

Chronic pain can be differentiated into various syndromes. One of these syndromes, which has been categorized as a subtype of neuropathic pain [for an overview see (13)], is complex regional pain syndrome (CRPS) which affects 4–7% of patients after limb injury (14, 15). After several weeks, patients develop chronic neuropathic pain in the affected limb that often includes somatosensory, motor and autonomic dysfunctions (16). Perceptual symptoms have been described which most frequently comprise impaired somatosensory discrimination (17), allodynia (18), neglect like symptoms (19), and a feeling of swelling of the affected limb [for a latest systematic observation (20)]. Motor dysfunction may involve dystonic movements (21), tremor, a reduced motion range and coordination deficits (22).

### GMV-Alterations in CRPS

Contradictory findings have been reported for GMV alterations in CRPS (23–27). Geha et al. (23) included 22 CRPS patients with upper and lower limb but also trunk affection and 22 healthy controls (HC) in a VBM analysis. Group differences were based on cluster thresholding after using permutation-based interference and was performed with FSL-scripts. ROI-analyses for linear regression were based on group differences. Overall, the slope of GMV-decrease with age was stronger in CRPS patients. Circumscribed GMV decrease for patients had been detected for the ventromedial PFC, anterior insula and the nucleus accumbens and this decrease was associated with years of persistence of CRPS (very heterogeneous patient group with 3 months to 13.5 years CRPS persistence) but also for the vmPFC with pain intensity [VAS (0–10); varied between 1.5 and 9.7].

Pleger et al. (24) applied VBM in 20 CRPS patients with unilateral upper limb affection and 1–63 months of CRPS duration (current pain intensity on a NRS from 1.5 to 8) and 20 age and gender matched controls. Group comparisons were based on cluster thresholding (FWE) after peak thresholding with *p* = 0.001. In addition, the authors applied a ROI-analysis for the pre- and postcentral gyrus. The results of this study were surprising since they found higher GMV for CRPS patients compared to HCs in dmPFC and M1 contralateral to the affected hand side. However, it is unknown how generalizable these findings are since some of the methods used in this study were older (1.5 T MRI, SPM/VBM8 used for GMV quantification, no total intracranial volume included as covariate for statistical analysis) and most patients (with 2 exclusions) were in the subacute stage of the disease.

Barad et al. (25) applied VBM in 15 right upper limb CRPS female patients and 15 matched HCs. Pain duration ranged from 2 months to 17 years and pain intensity was 7.5 of 10 (VAS) on average. The study used a 3 T MRI and non-isometric voxel size with unusual poor spatial resolution (28 slices; 4 mm slice thickness; 1 mm gap; in plane resolution 1.5 mm). The authors applied a FDR corrected threshold with *p* < 0.005; resulting in *t* = 3.71 threshold but only reported clusters with at least 30 voxels (both for group comparisons and linear regression with clinical data). The study found decreased GMV in the posterior insula, left OFC and CC (posterior ACC, posterior medial CC). However, an increase in GMV in bilateral putamen and right hypothalamus was also found. Higher pain intensity resulted in lower GMV in the dlPFC.

van Velzen et al. (26) did not observe any significant differences when comparing GMV in 19 upper limb patients (with right and left hand affection) and 19 matched HCs. The latest VBM-investigations on CRPS-patients (27) investigated 20 CRPS patients with affected right upper limbs. They performed elaborate sensorimotor testing with a focus on rigidity and dystonic symptoms, but also performed somatosensory testing, testing of autonomic function, and psychological testing. They applied state of the art MRI and VBM methods (3T imaging, SPM12 and CAT12, DARTEL based normalization, 5 mm smoothing) but performed a ROI-analysis restricted on basal ganglia, thalamus, insula, and postcentral gyrus. When compared to HCs only bilateral putamen showed relevant decrease in GMV. GMV reduction in the basal ganglia were associated with dystonic symptoms.

These divergent results of the aforementioned studies might well be caused by small sample sizes, inhomogeneous patient groups (lateralization/localization of the affected area, severity and/or duration of disease, type of CRPS), and differing or non-optimized evaluation strategies focusing on structural gray matter alterations. Overall, small sample sizes and large variances often prevent exploratory whole brain volume analyses and the necessary correction for multiple comparisons over said volume, favoring less conservative statistical approaches which are one cause of the reproducibility crisis in psychology [for the field of brain imaging see (28)]. By using a region of interest (ROI) driven approach any statistical effects detected are dependent on the ROIs defined. However, this definition is statistically only justified if based on previous investigations utilizing an exploratory whole brain approach, corrected for multiple comparisons over all measurements.

### Hypothesis and Methodological Approach

We here investigated common and specific GMV alterations in patients with CRPS from two study samples in an explorative (multiple comparison correction for the whole brain volume) and a hypothesis-driven approach (multiple comparison correction for regions of interest comprising mPFC/ACC, thalamus, and insula). The following hypotheses were tested:

- Explorative approach with correction over the whole brain volume and a ROI approach for those areas which already showed effects in previous investigations surviving a correction for multiple comparisons over the measurement volume: Are there GMV alterations in CRPS patients compared to healthy controls?
- ROI-approach for those areas which already showed effects in previous investigations surviving a correction for multiple comparisons over the measurement volume: Are there associations between GMV decrease in CRPS patients and pain severity, chronicity, and relevant performance impairment alterations in GMV?

## Methods

### Participants

Participants were recruited *via* support groups in Northern Germany and *via* the hand surgery and Anesthesiology of the University of Greifswald. Twenty-four right hand affected CRPS-patients have been included which have been characterized in detail in Table 1.

**Table 1:** Characteristics of CRPS patients.

**Participants**	**Age**	**CSS^a^**	**BDI^b^**	**Pain rest (VAS)**	**Duration (months)**	**Roeder_aff^c^**	**Two point discrimination (TPD)**	**Medication^d^**
Subject 1	56	17	7	2.5	14	250	8.0	A, B, D
Subject 2	48	15	35	5.0	14	117	3.0	A, B, C, D
Subject 3	65	–	3	8.0	104	250	3.0	–
Subject 4	60	9	14	5.5	72	80	3.2	D
Subject 5	24	11	22	4.5	84	48	3.7	A, B, D
Subject 6	47	10	11	4.5	22	51	1.7	B, D
Subject 7	19	13	6	0.0	12	76	1.9	B, C, D
Subject 8	53	14	14	7.0	26	250	10.0	B, D
Subject 9	58	13	3	4.5	61	104	1.5	B, C, D
Subject 10	64	8	20	2.5	13	65	1.8	B, C, D
Subject 11	44	9	6	9.5	77	81	3.5	B
Subject 12	79	5	17	10.0	50	106	3.3	–
Subject 13	57	12	7	3.0	84	95	3.7	A, B, D
Subject 14	19	13	32	2.0	48	74	2.7	B, C, D
Subject 15	33	11	13	5.0	51	109	3.00	A, B, C
Subject 16	56	13	22	10.0	106	50	3.3	A, B, D
Subject 17	59	16	16	3.0	95	84	3.7	B, C, D
Subject 18	64	13	10	2.0	74	48	2.7	A, B, C, D
Subject 19	54	13	10	5.0	109	51	3.0	A
Subject 20	61	13	–	1.5	4	38	4.4	A
Subject 21	40	16	–	2.4	8	84	7.0	A, C
Subject 22	58	7	–	8.1	5	63	4.0	A
Subject 23	45	14	–	7.9	15	250	10.0	A
Subject 24	55	6	–	2.7	7	51	4.6	–
Average ± SD	50.8 ± 14	11.8 ± 3.2	14.0 ± 8.9	4.8 ± 2.9	48 ± 37	103 ± 70	4.0 ± 2.4	

a *CSS, CRPS severity score (29)*.

b *BDI, Beck-Depression-Inventory (30)*.

c *Roeder_aff, Roeder test for the affected hand side*.

d *A: NSAID, non-steroidal anti-inflammatory drugs; B: opioid; C: amitriptyline; D: pregabalin*.

Twenty-four patients had been diagnosed based on the Budapest criteria (31) were on average 50.75 ± 14 years old, with 4 male, 17 right handed (assessed using the Edinburgh Handedness score) (32), CRPS-severity with CSS (CRPS severity score) (33) was 11.78 ± 3.23 on average, duration of symptoms on average for 48.12 ± 37 months, average rest pain was 4.80 ± 2.82 (VAS 0–100). Thirty-three healthy controls (25 right handed) were recruited by advertising from the University Medicine faculty with 54.42 ± 13.49 years old on average (sex not matched since these were 14 male participants). All participants were free from other neurological and psychiatric problems as assessed by a neurologist (ML). All participants gave their written informed consent. The two study samples have been approved by the local ethics committee of the University Medicine Greifswald (BB 45/09, BB 055/18).

### Sample Size Estimation

Sample size calculation was based on the first study published on GMV changes in CRPS patients (23). They included 22 CRPS patients and 22 HCs and described a cluster volume *p* < 0.05 effect for GMV decrease in the medial prefrontal cortex (mPFC). The mPFC appeared to be a robust finding in a number of different studies on associations of GMV decrease with pain chronicity; see also Kang et al. (34). Since the methods of determining GMV differences between samples were performed with other software packages we increased group size for both samples and included more homogeneous patients with respect to pain locations (upper limb, only dominant side). In a ROI-based approach for thalamus, ACC, mPFC, putamen, and insula we had to correct for false positive results within 39 resels (smoothed spatial units as the basis for the GLM). Therefore, we can expect a significant GMV-effect, based on results in other chronic pain groups, with a *t*-value of ≥4.1. Therefore, group sizes of our samples should be sufficient to test the aims as defined above.

### Assessments and Scores

Behavioral testing for the CRPS patients was performed in the same way as described before in previous investigations from our group [in healthy (35) in CRPS-patients (36)]. Two-point-discrimination (TPD) was tested using a wheel-discriminator (Sensidisk, Hannover, Germany) on fingertip of D1 in a pseudorandomized order of space intervals from 15 to 1 mm. Finger dexterity of the affected hand was assessed using the Roeder Manipulative Aptitude Test (Lafayette Instrument Company, Lafayette, IN, USA). In this test, the time needed to screw small rods into a row with ten holes was measured. Patients were asked for their current medication at the day when the imaging was performed (see Table 1).

### MRI-Measurements

MRI was performed with a 3 T Magnetom Verio (Siemens, Erlangen, Germany) using a 32-channel head coil. T1-weighted structural scans were acquired using the following characteristics: MP-RAGE, TR 1,690, TE 2.52 ms, flip angle 9°, matrix size 256 × 256, voxel size 1 × 1 × 1 mm^3^.

### MRI Evaluations

GMV alterations between all patients and controls were compared using CAT12/SPM12 packages. Statistical thresholding had been obtained in a generalized procedure for all comparisons: We will apply *p* < 0.05 (voxel height) using a family wise error (FWE) correction over (1) the whole brain, and (2) in an additional ROI-correction (FWE voxel height) comprising mPFC (one medial region), ACC (one medial region), and bilateral ROIs (anterior insula, thalamus, putamen). ROIs for GMV analyses were tested on the basis of prior investigations (23–27) and reports on GMV alterations in patients with neuropathic pain [e.g., (37)].

We aimed to identify differences in GMV between CRPS patients and age matched HCs. Furthermore, we investigated whether clinical symptoms (pain intensity and duration of CRPS) or sensorimotor performance (Roeder and TPD) were associated with GMV.

## Results

**Figure 1:**
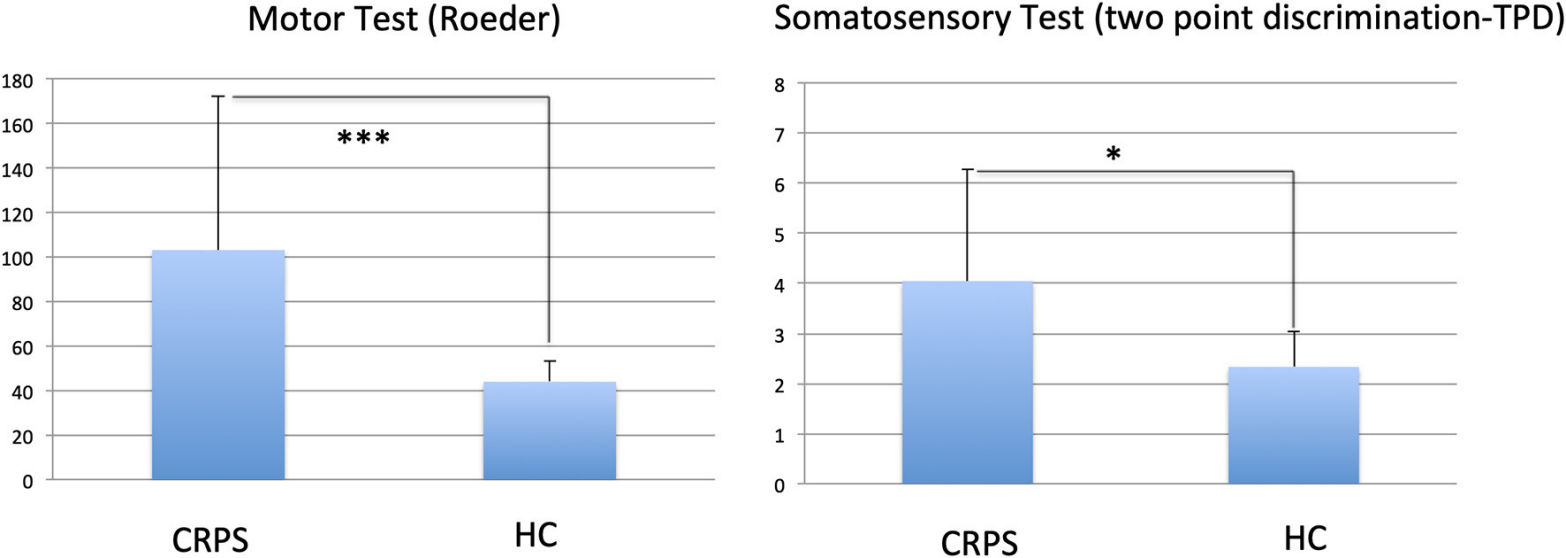
Comparison of motor (Roeder Test) and somatosensory (two point discrimination TPD) tests between CRPS patients and healthy controls (HC); stars indicate p-value of differences; ***0.001; *0.05. Larger values indicate lower performance.

CRPS patients showed decreased motor performance with their affected right hand than HCs (Roeder test; *t* = 3.24; *p* = 0.001). In addition, CRPS patients showed a decrease in spatiotactile discrimination as tested with the two-point discrimination (TPD; *t* = 2.91; *p* = 0.012; see Figure 1).

Pain intensity at rest, duration of CRPS symptoms, and performance were not relevantly associated with GMV alterations (for rest pain associations: duration: *r* = 0.32; n.s.; Roeder: *r* = 0.28; n.s.; TPD: *r* = 0.15; n.s.).

**Figure 2:**
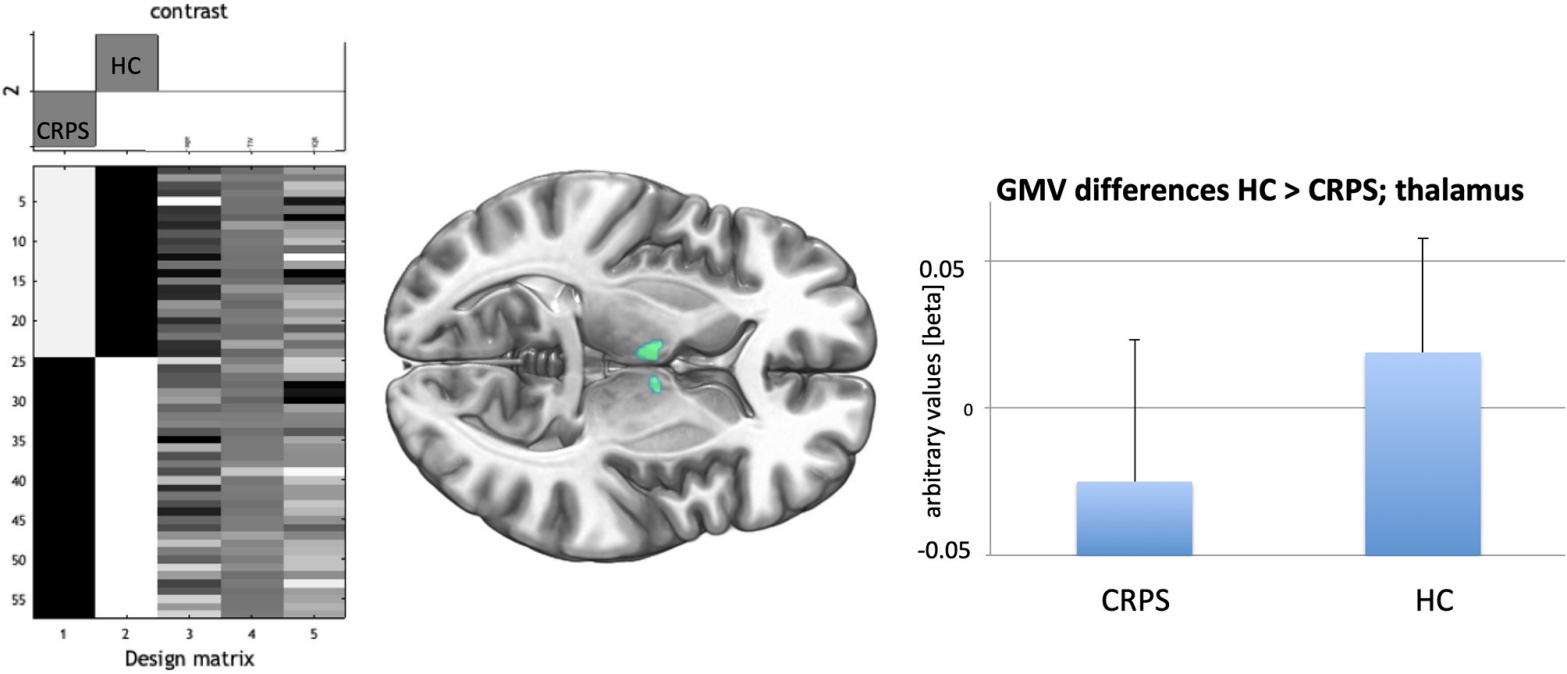
**(Left)** Design matrix of the t-test comparison CRPS (−1) against HC (+1) with the three covariates age, total intracranial volume, and a quality score/IQR derived from CAT12 preprocessing. **(Middle)** Bilateral decreased GMV (*p*_FWE_ < 0.05; cluster level) in the medio-anterior thalamus for the CRPS patients indicated in green. **(Right)** bars indicate averages together with SDs (lines) for the GMV-effect in the highest significant voxel of the medio-anterior thalamus for CRPS patients and HCs.

For the exploratory GMV analyses (corrected for the whole brain volume) CRPS patients showed lower GMV in the bilateral thalamus (cluster level whole brain correction: *t* = 4.42; 1,227 voxels; *p*_FWE_ = 0.015) than HCs (Figure 2). In particular, the strongest effects were detected in the thalamus proper (Neuromorphometrics brain atlas) for both hemispheres (left: *t* = 4.42; coordinates: −2, −10, 17; right: *t* = 4.03; coordinates: 9, −4, 11). For the ANATOMY/Oxford atlas differentiation (based on connectivity information) the temporal (right: *t* = 4.32; coordinates: 3, −10, 14) and the prefrontal (left: *t* = 3.67; coordinates: −6, −9, 6) parts of the thalamus showed highest effects. Effects remained largely unchanged when only including patents with more than 12 months of disease persistence for the group comparisons (CRPS: *n* = 20; left thalamus (coordinates: −5, −7, 8); *t* = 4.26; cluster level *p* = 0.008).

When using ROI-analysis (bilateral thalamus mask) we again only observed bilateral anterior-medial thalamus GMV decrease for CRPS (ri: coordinates: 3, −11, 16; *t* = 4.26; *p*_FWE_ = 0.008; le: coordinates: −3, −14, 14; *t* = 4.14; *p*_FWE_ = 0.011) but not for the other ROIs selected.

**Figure 3:**
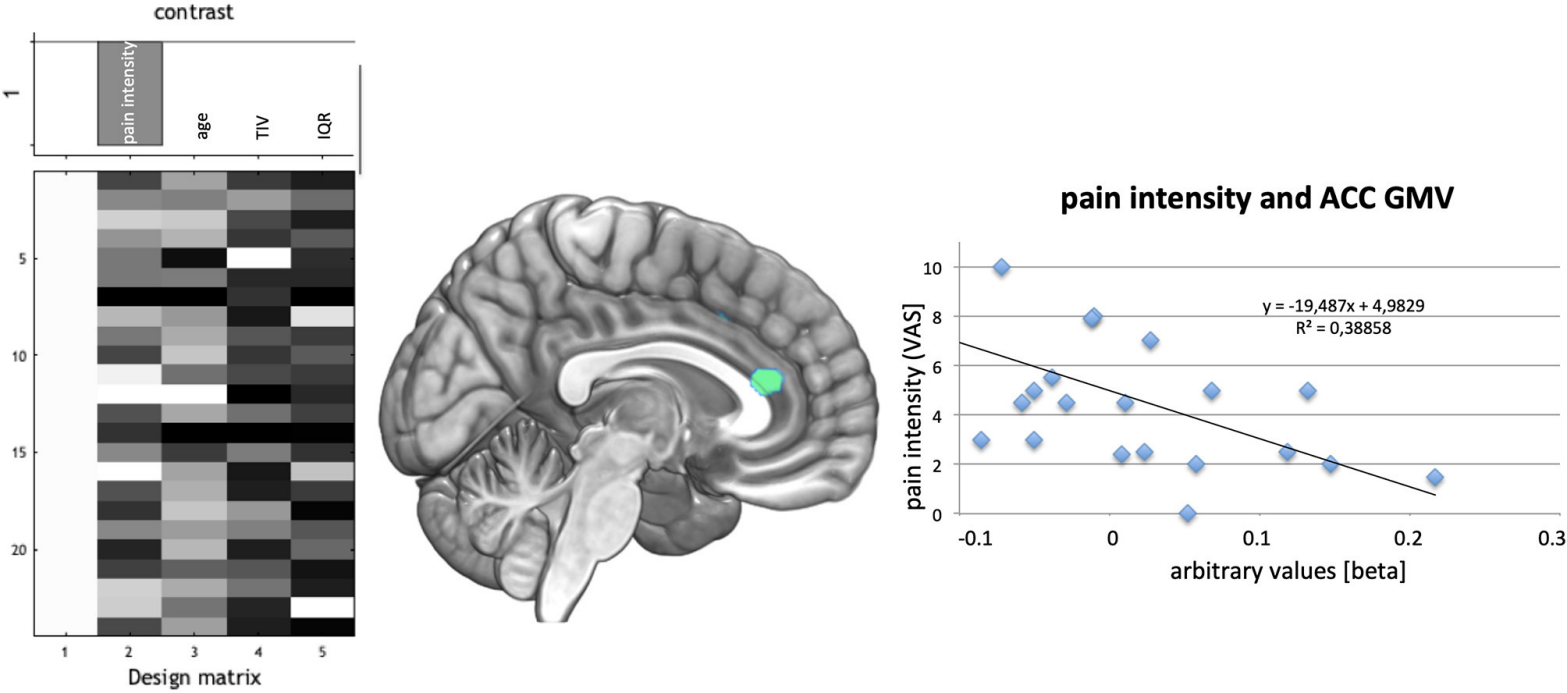
**(Left)** Design of regression analysis in the CRPS patient group for the factor pain intensity during rest using age, total intracranial volume (TIV), and quality of T1-images for segmentation (IQR) as covariates. **(Middle)** Association of pain during rest with GMV decrease in the cingulate cortex- indicated green. **(Right)** Plot of the association of decreased GMV in the highest significant voxel with pain intensity showed an *r*^2^ of 0.388.

Linear regression analyses for duration of CRPS revealed a decrease in GMV for the ACC (coordinates: −6, 31, 18; *t* = 4.07, *p*_FWE_ = 0.033; one sided). Pain intensity was negatively associated with GMV in the same Area (ACC; coordinates: 3, 34, 15; *t* = 4.23, *p*_FWE_ = 0.026; one sided; see Figure 3).

When testing possible associations between decrease in GMV with sensorimotor performance we used those parameters which were impaired in our patients compared to healthy controls. For the motor testing with the Roeder test we found a negative association with GMV in the left posterior insula [MNI coordinates: −42, −12, 9; *t* = 4.49; *p*_FWE_ (insula ROI) = 0.038]. For the somatosensory testing with TPD we observed a negative association with GMV in the left posterior insula [MNI coordinates: −44, −13, 6; *t* = 4.40; *p*_FWE_ (insula ROI) = 0.047].

## Discussion

By using voxel-based morphometry to compare gray matter volume of a highly homogeneous CRPS patient sample with those of healthy age matched controls we found a decrease for the patients GMV in the medial parts of the thalamus. This finding is analogous to other studies comparing GMV in neuropathic pain patients with those of healthy volunteers (9). It also matches well to reports on altered functional thalamo-cortical interaction in patients with neuropathic pain (38) and to models describing interactions of thalamic GMV decrease, a decrease of thalamic inhibitory neurotransmitters, and increased cortical excitability (37). Furthermore, anterior cingulate cortex GMV was negatively associated with duration of CRPS and pain intensity. This finding supports the results of others who showed associations of persistence of CRPS and GMV (23), and showed a decrease in ACC GMV in patients with other neuropathic pain syndromes [trigeminal neuralgia (8, 10)], or patients with milder non-neuropathic pain syndromes (11, 12). In addition, somatosensory performance impairment in CRPS patients was associated with lower GMV in the posterior left insula cortex. Although many studies already observed insula GMV decrease in patients with chronic neuropathic pain [trigeminal neuralgia (8, 10); herpes zoster (39); burning mouth syndrome (40)] and non-neuropathic pain [e.g., CBP (12)] no such associations with sensorimotor performance have yet been described.

### Thalamus Effect

In the present VBM investigation in a group of carefully selected patients with upper limb affection of the dominant right hand we found decreased GMV in comparison to matched healthy controls in the bilateral thalamus proper. This effect remained largely unchanged after excluding all patients with <1 year of disease persistence, underlining the observation that GMV decrease is related to persistence of chronic pain [e.g., (23)]. Compared to a connectivity-based atlas this resembles the areas connected to the prefrontal and temporal cortex. Especially for the prefrontal interactions these parts of the thalamus might be related to both pain modulation [discussion see (41)] or effects of the ACC [e.g., (11)] which had been described to be vulnerable to both chronic pain but also stressors in animal research before (42, 43). Especially the thalamo-prefrontal axis is an important hub which shows changes in cholinergic neurotransmitters in chronic pain patients (41) and specifically a decrease connectivity for CRPS patients (38). At least with respect to the ACC GMV decrease it seems to be highly associated with the duration of CRPS. For the temporal areas this might well be related also to parts of the limbic system known to be involved in the modulation of pain intensity [for animal literature (44)].

An overlapping area in the ventroposterior thalamus has been described to be reduced in GMV before, showing reduced connectivity and reduced GABA for patients with neuropathic pain [for trigeminal neuralgia (9, 37)].

Interestingly, Fukumato et al. used radioactive labeled iodoamphetamine in a SPECT study in 10 patients with CRPS to investigate perfusion differences between the hemispheres contra- and ipsilateral to the affected upper limb. When compared to ipsilateral, contralateral thalamus showed a reduction in perfusion and this reduction index was related to time of onset of the disease (6–36 months). In contrast, healthy controls showed symmetric thalamic perfusion. They discussed their results with the finding that chronic neuropathic pain results in a long-term thalamic inhibition whereas acute pain increases its activity. In contrast to Fukumoto, we observed a bilateral reduction in thalamic GMV. However, the effect for the contralateral hemisphere to the affected hand was stronger—especially when investigating only patients who had more than 12 months duration of CRPS.

### ACC Effect

Here, the anterior cingulate cortex GMV was negatively associated with the duration of CRPS and pain intensity. This finding supports finding of others who showed associations of persistence of CRPS and GMV (23), showed a decrease in ACC GMV in patients with other neuropathic pain syndromes [trigeminal neuralgia (8, 10)], or patients with milder non-neuropathic pain syndromes (11, 12). In a monkey model the anterior cingulate cortex and the medial PFC showed that reduced GMV was associated with stress (42). In addition, cognitive deficit, e.g., for attention, was associated with a decrease in ACC/mPFC GMV in fibromyalgia (45). Neuropathic pain, increased stress, sleeplessness, attention deficits, and decrease in prefrontal pain suppression might well contribute to the maladaptive chronification circle into sustained pain.

### Insula Effect

In the rodent neuropathic pain model using nerve compression induced by surgical intervention S1, ACC, and insula GMV loss was associated with somatosensory impairment (43). The insula serves as an internal monitor adjusting all incoming input into a current body state. The anterior insula is densely interconnected with the prefrontal cortex and the limbic system (46), it is therefore part of the emotional/anticipation pain system. In contrast, the posterior insula is highly interconnected with the thalamus and the somatosensory cortices (S1 and S2) and is therefore part of the somatosensory discriminative pain processing system. Hence, it is not surprising that a reduction in gray matter volume in this area is associated with somatosensory performance such as spatiotactile resolution (as tested with the TPD) but also with pinch grip performance (as tested with the Roeder test). Prior studies have demonstrated associations between pinch grip, motor and TPD testing in CRPS patients (17). Several studies already observed insula GMV decrease in patients with chronic neuropathic pain [trigeminal neuralgia (8, 10); herpes zoster (39); burning mouth syndrome (40)] and non-neuropathic pain [e.g., CBP (12)]. In contrast to Geha et al., who found an association of GMV-decrease with duration of CRPS for the anterior insula, we here observed effects for the posterior insula. Overall, the anterior insula activity had been identified to be associated with catastrophizing pain and trait anxiety in other groups of chronic pain patients (TMD) (47), and its increased activation in chronic pain patients decreases following interventions successfully reducing pain intensity (48). When considering its connections to the prefrontal lobe an association of persistence of the pain syndrome in the Geha et al. (23) study and our finding of an association of posterior insula GMV with a somatosensory performance is in a good concordance with anatomical connectivity of this area.

### What Else Could Be the Basis of GMV-Alterations in Chronic Pain?

There is an ongoing debate concerning the fundamental neural substrates of GMV alterations. Underlying mechanisms for GMV decrease in patients with chronic pain have been discussed recently on the basis of ACC/mPFC GMV (34). One of the rare longitudinal studies examined patients who underwent hip surgery suffering chronic pain due to hip osteoarthritis. After the intervention the patients were pain free and the study authors suggested that VBM changes are a consequence but not the cause of pain (49). Research in chronic pain models in animals have provided insight into the underlying mechanisms of GMV decrease in the presence of chronic pain [e.g., (42)]. Neuropathic pain, using nerve compression models in rodents, revealed a loss of GMV after surgery in the primary somatosensory cortex (S1), ACC and insula that was associated with a decrease in somatosensory performance (43). Taken together, animal studies showed that (1) prefrontal GMV loss is causally related to surgical intervention resulting in chronic neuropathic pain in rodents, and (2) S1, ACC, and insula GMV loss is associated with somatosensory impairment induced by neuropathic pain as a result of a surgical intervention.

### Methodological Considerations

When reviewing the literature on GMV alterations in CRPS it is very difficult to summarize common findings. One important reason might be the inhomogeneous patient cohorts investigated before. Geha et al. (23) included upper limb, lower limb and trunk localization of CRPS. Inhomogeneity in CRPS-affection and symptoms increase noise and more recent studies (27) focused on patients with right upper limb affection. In addition, the stage of CRPS is crucial since persistence of the pain syndrome has been associated with GMV loss [for non-neuropathic pain see for instance (11)]. Pleger et al. (24) predominantly included CRPS patients in the subacute stage and tested them against healthy controls. Their surprising finding of an increase in GMV in areas which show a clear GMV decrease in most other studies might well be related to that inclusion criteria. Furthermore, most samples were clearly underpowered when considering effect sizes resulting from larger samples of chronic pain patients [e.g., (11, 12)]. On the measurement side, a high resolution isotropic spatial resolution (at least 1 mm^3^) should be standard nowadays. Resampling 4 mm slice thickness with 1 mm spacing between slices (gap) into a voxel-based morphometry is not even justifiable when considering technical deficits of past decades (25). It is also essential on the measurement side, that only data derived from the same MRI should be included in the group analysis or advanced harmonization approaches are used prior to analysis. In addition, the latest data processing tools for GMV-analysis provide state-of-the art approaches regarding preparation, artifact reduction, segmentation, advanced normalization procedures and quality assessment (e.g., CAT—A computational anatomy toolbox for the analysis of structural MRI data; C Gaser, R Dahnke; OHBM 2016, 4057) which is extremely important for accuracy of the VBM method. Last, but not least, a reliable statistical approach is essential: a severe problem in the field is the restriction of analysis on regions of interest, especially if these have not stood the proof of an exploratory whole brain corrected analysis before as performed by Azqueta-Gavaldon et al. (27). It has also to be mentioned that for the highly important longitudinal studies on chronic pain and GMV alterations the current software packages do not offer reliable and comparable solutions leaving some necessary methodological work on standardized procedures for trustworthily detecting longitudinal changes in GMV.

### Limitations

Studies on diseases with fortunately rare occurrence always lack from low statistical power. However, this study currently is based on the largest and most homogeneous sample of CRPS patients and healthy controls. However, we did not equally balance control participants for gender but controlled for TIV as a nuisance variable in our statistical design. However, we did not lodge and lock our protocol and statistical analysis plan prior to commencing data collection. It was not commonplace to do so when we started this study, but now it is recommended (50). Failure to do this clearly represents a shortcoming in transparency and reporting. We here decided to follow a voxel-wise GMV estimation to detect local changes within the brain between groups which also enabled a whole-brain statistical analyses approach. We admit that ROI-based comparisons between groups for the whole thalamus might show different effects since they are not testing local changes within the structure. Although we carefully documented the current medication at the time point of MRI-investigation (see Table 1) patients had a long-lasting history of all kind of interventions and these might well have an impact on our findings. In addition, we did not score mood disorders over all patients and therefore cannot exclude that depression might have had an impact on our findings. However, in a new ALE meta-analysis comprising 46 VBM studies on mood disorders the anterior insula predominantly showed a reduction in GMV (51). Therefore, GMV reduction of the bilateral thalamus induced by mood disorder, which is more frequent for patients with chronic pain syndromes than in healthy subjects, might be improbable.

## Conclusion

In conclusion, we here present a VBM analysis on a considerable sample of CRPS patients in the chronic stage of the disease. In contrast to studies of non-neuropathic chronic pain, which primarily report a loss of GMV in ACC and the insula, our patients predominantly showed medial thalamic GMV loss. Additionally, regression analyses with pain intensity and duration identified a negative association with ACC GMV. Furthermore, somatosensory impairment was associated with GMV loss in the insula, until now a finding in rodent neuropathic pain models, here for the first time shown in a human sample of patients with chronic neuropathic pain.

## Data Availability Statement

The raw data supporting the conclusions of this article will be made available by the authors, without undue reservation.

## Ethics Statement

The studies involving human participants were reviewed and approved by the local ethics committee of the University Medicine Greifswald (BB 45/09, BB 055/18). The patients/participants provided their written informed consent to participate in this study.

## Author Contributions

SS organized patient recruitment and corrected the manuscript. MD helped with data evaluation and corrected the manuscript. JM corrected the manuscript and added ideas in data evaluation. ML managed the study, evaluated data, wrote the manuscript, and corrected and submitted the manuscript. All authors contributed to the article and approved the submitted version.

## Funding

ML and JM were supported by the Australian National Health and Medical Research Council (NHMRC) grant #1163149. SS was awarded a Gerhard-Domagk fellowship for undertaking this study; he also received a grant by the Else Kröner Fresenius-Stiftung 2019-A24. In addition, the investigations of GMV in chronic pain are subject to a funding of the DFG for ML (LO 795/37-1).

## Conflict of Interest

The authors declare that the research was conducted in the absence of any commercial or financial relationships that could be construed as a potential conflict of interest.

## Publisher's Note

All claims expressed in this article are solely those of the authors and do not necessarily represent those of their affiliated organizations, or those of the publisher, the editors and the reviewers. Any product that may be evaluated in this article, or claim that may be made by its manufacturer, is not guaranteed or endorsed by the publisher.

## References

[1] van Hecke O Torrance N Smith BH. Chronic pain epidemiology and its clinical relevance. Br J Anaesth. (2013) 111:13–8. 10.1093/bja/aet123 23794640

[2] Phillips K Clauw DJ. Central pain mechanisms in chronic pain states–maybe it is all in their head. Best Pract Res Clin Rheumatol. (2011) 25:141–54. 10.1016/j.berh.2011.02.005 22094191 PMC3220875

[3] Cauda F Palermo S Costa T Torta R Duca S Vercelli U. Gray matter alterations in chronic pain: a network-oriented meta-analytic approach. Neuroimage Clin. (2014) 4:676–86. 10.1016/j.nicl.2014.04.007 24936419 PMC4053643

[4] Lin C Lee SH Weng HH. Gray matter atrophy within the default mode network of fibromyalgia: a meta-analysis of voxel-based morphometry studies. Biomed Res Int. (2016) 2016:7296125. 10.1155/2016/7296125 28105430 PMC5220433

[5] Baliki MN Geha PY Apkarian AV Chialvo DR. Beyond feeling: chronic pain hurts the brain, disrupting the default-mode network dynamics. J Neurosci. (2008) 28:1398–403. 10.1523/JNEUROSCI.4123-07.2008 18256259 PMC6671589

[6] Schmidt-Wilcke T Leinisch E Gänssbauer S Draganski B Bogdahn U Altmeppen J. Affective components and intensity of pain correlate with structural differences in gray matter in chronic back pain patients. Pain. (2006) 125:89–97. 10.1016/j.pain.2006.05.004 16750298

[7] Apkarian AV Sosa Y Sonty S Levy RM Harden RN Parrish TB. Chronic back pain is associated with decreased prefrontal and thalamic gray matter density. J Neurosci. (2004) 24:10410–5. 10.1523/JNEUROSCI.2541-04.2004 15548656 PMC6730296

[8] Obermann M Rodriguez-Raecke R Naegel S Holle D Mueller D Yoon MS. Gray matter volume reduction reflects chronic pain in trigeminal neuralgia. Neuroimage. (2013) 74:352–8. 10.1016/j.neuroimage.2013.02.029 23485849

[9] Gustin SM Peck CC Wilcox SL Nash PG Murray GM Henderson LA. Different pain, different brain: thalamic anatomy in neuropathic and non-neuropathic chronic pain syndromes. J Neurosci. (2011) 31:5956–64. 10.1523/JNEUROSCI.5980-10.2011 21508220 PMC6632967

[10] Wang Y Cao DY Remeniuk B Krimmel S Seminowicz DA Zhang M. Altered brain structure and function associated with sensory and affective components of classic trigeminal neuralgia. Pain. (2017) 158:1561–70. 10.1097/j.pain.0000000000000951 28520647

[11] Domin M Grimm NK Klepzig K Schmidt CO Kordass B Lotze M. Gray matter brain alterations in temporomandibular disorder tested in a population cohort and three clinical samples. J Pain. (2021) 22:739–47. 10.1016/j.jpain.2021.01.003 33529707

[12] Fritz HC McAuley JH Wittfeld K Hegenscheid K Schmidt CO Langner S. Chronic back pain is associated with decreased prefrontal and anterior insular gray matter: results from a Population-Based Cohort Study. J Pain. (2016) 17:111–8. 10.1016/j.jpain.2015.10.003 26476265

[13] Dworkin SF Sherman J Mancl L Ohrbach R LeResche L Truelove E. Reliability, validity, and clinical utility of the research diagnostic criteria for temporomandibular disorders axis II scales: Depression, non-specific physical symptoms, and graded chronic pain. J Orofac Pain. (2002) 16:207–20. 12221737

[14] Beerthuizen A Stronks DL Van't Spijker A Yaksh A Hanraets BM Klein J. Demographic and medical parameters in the development of complex regional pain syndrome type 1 (CRPS1): prospective study on 596 patients with a fracture. Pain. (2012) 153:1187–92. 10.1016/j.pain.2012.01.026 22386473

[15] Birklein F Handwerker HO. Complex regional pain syndrome: how to resolve the complexity? Pain. (2001) 94:1–6. 10.1016/S0304-3959(01)00393-1 11576739

[16] Veldman PH Reynen HM Arntz IE Goris RJ. Signs and symptoms of reflex sympathetic dystrophy: prospective study of 829 patients. Lancet. (1993) 342:1012–6. 10.1016/0140-6736(93)92877-V 8105263

[17] Pleger B Ragert P Schwenkreis P Förster AF Wilimzig C Dinse H. Patterns of cortical reorganization parallel impaired tactile discrimination and pain intensity in complex regional pain syndrome. Neuroimage. (2006) 32:503–10. 10.1016/j.neuroimage.2006.03.045 16753306

[18] Maihöfner C Handwerker HO Birklein F. Functional imaging of allodynia in complex regional pain syndrome. Neurology. (2006) 66:711–7. 10.1212/01.wnl.0000200961.49114.39 16534108

[19] Galer BS Jensen M Butler S. Neglect-like signs and symptoms in CRPS. Pain. (2013) 154:961–2. 10.1016/j.pain.2013.02.024 23562169

[20] Reinersmann A Skinner IW Lücke T Massy-Westropp N Rudolf H Moseley GL. Intact tactile anisotropy despite altered hand perception in complex regional pain syndrome: rethinking the role of the primary sensory cortex in tactile and perceptual dysfunction. PeerJ. (2021) 9:e11156. 10.7717/peerj.11156 33986983 PMC8101475

[21] Birklein F Rowbotham MC. Does pain change the brain? Neurology. (2005) 65:666–7. 10.1212/01.wnl.0000179148.80687.f3 16157896

[22] Maihöfner C Baron R DeCol R Binder A Birklein F Deuschl G. The motor system shows adaptive changes in complex regional pain syndrome. Brain. (2007) 130(Pt 10):2671–87. 10.1093/brain/awm131 17575278

[23] Geha PY Baliki MN Harden RN Bauer WR Parrish TB Apkarian AV. The brain in chronic CRPS pain: abnormal gray-white matter interactions in emotional and autonomic regions. Neuron. (2008) 60:570–81. 10.1016/j.neuron.2008.08.022 19038215 PMC2637446

[24] Pleger B Draganski B Schwenkreis P Lenz M Nicolas V Maier C. Complex regional pain syndrome type I affects brain structure in prefrontal and motor cortex. PLoS One. (2014) 9:e85372. 10.1371/journal.pone.0085372 24416397 PMC3887056

[25] Barad MJ Ueno T Younger J Chatterjee N Mackey S. Complex regional pain syndrome is associated with structural abnormalities in pain-related regions of the human brain. J Pain. (2014) 15:197–203. 10.1016/j.jpain.2013.10.011 24212070 PMC4784981

[26] van Velzen GA Rombout SA van Buchem MA Marinus J van Hilten JJ. Is the brain of complex regional pain syndrome patients truly different? Eur J Pain. (2016) 20:1622–33. 10.1002/ejp.882 27161331

[27] Azqueta-Gavaldon M Youssef AM Storz C Lemme J Schulte-Göcking H Becerra L. Implications of the putamen in pain and motor deficits in complex regional pain syndrome. Pain. (2020) 161:595–608. 10.1097/j.pain.0000000000001745 31693538 PMC7179084

[28] Poldrack RA Baker CI Durnez J Gorgolewski KJ Matthews PM Munafò MR. Scanning the horizon: towards transparent and reproducible neuroimaging research. Nat Rev Neurosci. (2017) 18:115–26. 10.1038/nrn.2016.167 28053326 PMC6910649

[29] Harden RN Maihofner C Abousaad E Vatine JJ Kirsling A Perez RSGM. A prospective, multisite, international validation of the complex regional pain syndrome severity score. Pain. (2017) 158:1430–6. 10.1097/j.pain.0000000000000927 28715350

[30] Beck AT Steer RA Brown GK. Manual for the Beck Depression Inventory-II. San Antonio, TX: Psychol Corporation (1996).

[31] Harden RN Bruehl S Stanton-Hicks M Wilson PR. Proposed new diagnostic criteria for complex regional pain syndrome. Pain Med. (2007) 8:326–31. 10.1111/j.1526-4637.2006.00169.x 17610454

[32] Oldfield RC. The assessment and analysis of handedness: the Edinburgh inventory. Neuropsychologia. (1971) 9:97–113. 10.1016/0028-3932(71)90067-4 5146491

[33] Harden NR Bruehl S Perez RSGM Birklein F Marinus J Maihofner C. Development of a severity score for CRPS. Pain. (2010) 151:870–6. 10.1016/j.pain.2010.09.031 20965657

[34] Kang D McAuley JH Kassem MS Gatt JM Gustin SM. What does the grey matter decrease in the medial prefrontal cortex reflect in people with chronic pain? Eur J Pain. (2019) 23:203–19. 10.1002/ejp.1304 30101509

[35] Härtner J Strauss S Pfannmöller J Lotze M. Tactile acuity of fingertips and hand representation size in human Area 3b and Area 1 of the primary somatosensory cortex. Neuroimage. (2021) 232:117912. 10.1016/j.neuroimage.2021.117912 33652142

[36] Strauss S Grothe M Usichenko T Neumann N Byblow WD Lotze M. Inhibition of the primary sensorimotor cortex by topical anesthesia of the forearm in patients with complex regional pain syndrome. Pain. (2015) 156:2556–61. 10.1097/j.pain.0000000000000324 26270587

[37] Henderson LA Peck CC Petersen ET Rae CD Youssef AM Reeves JM. Chronic pain: lost inhibition? J Neurosci. (2013) 33:7574–82. 10.1523/JNEUROSCI.0174-13.2013 23616562 PMC6619566

[38] Di Pietro F Lee B Henderson LA. Altered resting activity patterns and connectivity in individuals with complex regional pain syndrome. Hum Brain Mapp. (2020) 41:3781–93. 10.1002/hbm.25087 32510695 PMC7416050

[39] Liu J Gu L Huang Q Hong S Zeng X Zhang D. Altered gray matter volume in patients with herpes zoster and postherpetic neuralgia. J Pain Res. (2019) 12:605–16. 10.2147/JPR.S183561 30799946 PMC6369852

[40] Lee YC Jahng GH Ryu CW Byun JY. Change in gray matter volume and cerebral blood flow in patients with burning mouth syndrome. J Oral Pathol Med. (2019) 48:335–42. 10.1111/jop.12838 30735586

[41] Kummer KK Mitrić M Kalpachidou T Kress M. The medial prefrontal cortex as a central hub for mental comorbidities associated with chronic pain. Int J Mol Sci. (2020) 21:3440. 10.3390/ijms21103440 32414089 PMC7279227

[42] Magariños AM McEwen BS Flügge G Fuchs E. Chronic psychosocial stress causes apical dendritic atrophy of hippocampal CA3 pyramidal neurons in subordinate tree shrews. J Neurosci. (1996) 16:3534–40. 10.1523/JNEUROSCI.16-10-03534.1996 8627386 PMC6579123

[43] Seminowicz DA Laferriere AL Millecamps M Yu JS Coderre TJ Bushnell MC. MRI structural brain changes associated with sensory and emotional function in a rat model of long-term neuropathic pain. Neuroimage. (2009) 47:1007–14. 10.1016/j.neuroimage.2009.05.068 19497372 PMC4486383

[44] McIlwrath SL Montera MA Gott KM Yang Y Wilson CM Selwyn R. Manganese-enhanced MRI reveals changes within brain anxiety and aversion circuitry in rats with chronic neuropathic pain- and anxiety-like behaviors. Neuroimage. (2020) 223:117343. 10.1016/j.neuroimage.2020.117343 32898676 PMC8858643

[45] Luerding R Weigand T Bogdahn U Schmidt-Wilcke T. Working memory performance is correlated with local brain morphology in the medial frontal and anterior cingulate cortex in fibromyalgia patients: structural correlates of pain-cognition interaction. Brain. (2008) 131(Pt 12):3222–31. 10.1093/brain/awn229 18819988

[46] Dupont S Bouilleret V Hasboun D Semah F Baulac M. Functional anatomy of the insula: new insights from imaging. Surg Radiol Anat. (2003) 25:113–9. 10.1007/s00276-003-0103-4 12819943

[47] Dammann J Klepzig K Schenkenberger E Kordass B Lotze M. Association of decrease in insula fMRI activation with changes in trait anxiety in patients with craniomandibular disorder (CMD). Behav Brain Res. (2020) 379:112327. 10.1016/j.bbr.2019.112327 31697982

[48] Lickteig R Lotze M Kordass B. Successful therapy for temporomandibular pain alters anterior insula and cerebellar representations of occlusion. Cephalgia. (2013) 33:1248–57. 10.1177/0333102413491028 23771211

[49] Rodriguez-Raecke R Niemeier A Ihle K Ruether W May A. Brain gray matter decrease in chronic pain is the consequence and not the cause of pain. J Neurosci. (2009) 29:13746–50. 10.1523/JNEUROSCI.3687-09.2009 19889986 PMC6666725

[50] Lee H Lamb SE Bagg MK Toomey E Cashin AG Moseley GL. Reproducible and replicable pain research: a critical review. Pain. (2018) 159:1683–9. 10.1097/j.pain.0000000000001254 29697535

[51] Jabbi M Arasappan D Eickhoff SB Strakowski SM Nemeroff CB Hofmann HA. Neuro-transcriptomic signatures for mood disorder morbidity and suicide mortality. J Psychiatr Res. (2020) 127:62–74. 10.1016/j.jpsychires.2020.05.013 32485434

